# Techno‐Functional Properties and Antioxidant Activities of Lactose‐Hydrolyzed Stirred Yoghurts Fortified With Hawthorn Fruit

**DOI:** 10.1002/fsn3.71729

**Published:** 2026-04-04

**Authors:** Muhammed Fidan, Canan Altınay, Halil İbrahim Öztaş, Samet Dönmez, Tuba Şanlı

**Affiliations:** ^1^ Graduate School of Natural and Applied Science Ankara University Ankara Turkey; ^2^ Department of Dairy Technology, Faculty of Agriculture Ankara University Ankara Turkey

**Keywords:** fortification, functional yoghurt, hawthorn fruit, lactose hydrolysis, rheological properties, turmeric powder

## Abstract

Stirred‐type yoghurts produced from lactose‐hydrolyzed milk represent a promising base for developing functional dairy products enriched with fruits and spices, as such formulations can enhance nutritional, sensory, and antioxidant properties while offering naturally sweetened alternatives without added sugars. Despite this potential, the incorporation of wild fruits with high bioactive potential into lactose‐free yoghurt systems presents both technological and sensory challenges, particularly regarding texture stability, color development, and consumer acceptance. This study evaluated the effects of enriching stirred‐type yoghurts produced from lactose‐hydrolyzed milk with varying levels of hawthorn fruit puree (5%, 10%, and 15%) and turmeric powder (0.1%) on their physicochemical, rheological, sensory properties, and antioxidant activities. Yoghurt samples were stored for 21 days under refrigerated conditions, and analyses were conducted at 7‐day intervals throughout storage. Yoghurts were formulated with increasing concentrations of hawthorn fruit puree and turmeric powder, and physicochemical parameters, water‐holding capacity, rheological and textural properties, antioxidant activities (DPPH and ABTS), color (hue angle), and sensory acceptance were assessed across treatments (YH5, YH10, YH15). Increasing amounts of hawthorn fruit puree significantly improved water‐holding capacity, while textural and rheological analyses indicated that the 10% level exhibited improved rheological behavior, particularly in viscosity, and maintained comparable gel structure during storage. Antioxidant capacities ranged from 58.9%–83.9% for DPPH and 3.12–5.89 mg TE/g for ABTS. YH5 exhibited the highest ABTS activity (*p* < 0.05), whereas its maximum DPPH activity was not statistically different from the control (*p* > 0.05). Hue angle values remained relatively stable during storage, with only minor fluctuations observed in some samples, indicating that the characteristic color tones associated with turmeric powder addition were largely preserved throughout the storage period. Sensory evaluations revealed that the YH10 sample was the most preferred by panelists. Overall, these findings highlight the potential of producing fruit‐ and spice‐enriched functional fermented dairy products from lactose‐hydrolyzed milk without added sugars, offering both nutritional and sensory benefits.

## Graphical abstract

This graphical abstract illustrates the production workflow of lactose‐hydrolyzed stirred yoghurts fortified with hawthorn fruit puree (5%, 10%, 15%) and turmeric, alongside the evaluation of their physical and sensory properties over a 21‐day storage period.

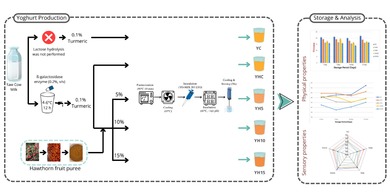

## 1 Introduction

The demand for nutritious and functional foods has increased substantially, and dairy products, which play a key role in healthy and balanced nutrition, have gained a significant market share in this field. Fermented milk products constitute a major segment of functional dairy foods and are globally recognized for their health‐promoting properties (Kanat et al. 2023; Dimitrellou et al. 2025). Among them, yoghurt is widely appreciated for its high nutritional value, particularly due to the bioavailability and digestibility of its proteins, calcium, and essential nutrients (Şanli 2015). In addition, yoghurt serves as an effective matrix for the development of value‐added functional products (Jany et al. 2024; Priyashantha et al. 2025). Numerous studies have focused on enhancing the functional properties of yoghurt through the incorporation of fruits, spices, or their combinations (Feng et al. 2019; Carmona et al. 2021; Ruikar et al. 2024; Wardani et al. 2023).

In the dairy industry, this innovative approach has been adopted, leading to the development and commercialization of various functional dairy products. While commonly used fruits dominate the market, wild fruits attract increasing attention due to their high levels of vitamins, minerals, and bioactive compounds such as carotenoids and phenolics (Trichopoulou et al. 2000; Herrera et al. 2023; Wang et al. 2025). Accordingly, the incorporation of wild fruits into fermented dairy products may serve not only to enhance product functionality but also to introduce underutilized natural resources into value‐added food systems.

However, developing functional dairy products remains challenging, as improvements in health‐related attributes must be achieved without compromising sensory quality. Maintaining desirable texture, flavor, and visual properties is therefore essential for consumer acceptance (Dimitrellou et al. 2025). Recent consumer analyses further indicate that healthy eating, freshness, taste, quality, and natural ingredients are among the primary factors influencing purchasing decisions (Statista 2024). In this context, achieving a balance between functional enhancement and technological–sensory performance represents a critical objective in yoghurt fortification studies.

In this study, the use of hawthorn fruit ( *Crataegus monogyna* L.), which grows naturally in the wild and is mostly known at a local level, was investigated in yoghurt production. Owing to its high antioxidant capacity, hawthorn fruit emerges as a promising natural ingredient for product fortification (Herrera et al. 2023; Utebaeva et al. 2024; Taneva and Zlatev 2024; Wang et al. 2025). The antioxidant compounds present in hawthorn fruit have been reported to support cardiovascular health, while its high dietary fiber content contributes to improved digestive function, and its rich bioactive profile strengthens the immune system (Nazhand et al. 2020; Cakmak 2024; Şeker et al. 2025). Moreover, the high pectin content of hawthorn fruit suggests its potential to enhance viscosity and emulsification properties in food products (Wang et al. 2025). However, due to the hardness and multi‐seeded structure of the fruit, direct consumption is difficult; therefore, processing it into puree and incorporating it into other products offers a more practical consumption route. Enrichment of dairy products with fruit purees may lead to color changes as a result of various chemical reactions (Ścibisz et al. 2019). To overcome this problem, different spices are used in combination with various fruit combinations (Priyashantha et al. 2025; Wardani et al. 2023; Wardani and Chamidah 2023). In our research, turmeric powder was selected to improve the color properties of the product while also taking into account the increasing consumer demand for foods with natural ingredients. Turmeric ( *Curcuma longa* ) has been reported to enhance digestion, regulate blood glucose levels, and assist in reducing abdominal fat mass (Buniowska‐Olejnik et al. 2024). As one of the most commonly used spices in foods, turmeric functions as a natural pigment, thereby increasing the visual appeal and sensory acceptability of the products in which it is used (El‐Shazly et al. 2011; Okur 2023; Buniowska‐Olejnik et al. 2024).

With increasing consumer health awareness, lactose‐free dairy products have emerged as an important category within functional dairy foods. Lactose intolerance is defined as a sensitivity that occurs when the body is unable to produce sufficient amounts of the enzyme lactase, leading to undigested lactose passing into the colon and causing symptoms such as diarrhea and gas formation (Pereira et al. 2020). Considering that approximately 70% of the global population is lactose intolerant (Facioni et al. 2020), the rising consumer awareness regarding lactose‐free dairy products has contributed to the rapid growth of this market within the dairy industry. It has been reported that the global lactose‐free dairy products market is projected to reach 40.73 billion USD by 2032, with lactose‐free yoghurt accounting for approximately 26.15% of this market in 2025 (Gusain 2024). Lactose‐free yoghurt not only meets the dietary needs of individuals with lactose intolerance but also has been reported to improve the sensory and rheological properties of the product through lactose hydrolysis (Ibrahim 2018; Pachekrepapol et al. 2021).

In this study, yoghurts were produced from lactose‐hydrolyzed milk with the addition of varying concentrations (5%, 10%, and 15%) of hawthorn fruit puree and a predetermined level of turmeric powder established through preliminary trials. Over a 21‐day storage period, with analyses conducted at 7‐day intervals, the physicochemical properties (pH, color, water‐holding capacity, and texture), rheological parameters (shear viscosity, flow behavior index, and consistency index), sensory characteristics, total phenolic contents, and antioxidant activities of the samples were evaluated. The aim was to develop a novel yoghurt variety enhanced with natural ingredients, offering improved functional properties for health‐conscious consumers and individuals with lactose intolerance, while maintaining sensory qualities that meet consumer acceptance.

## 2 Materials and Methods

### 2.1 Materials

Raw cow's milk standardized to 2.5% fat was obtained from the Pilot Dairy Processing Plant of Ankara University, Faculty of Agriculture (Ankara, Türkiye) for yoghurt production. The chemical composition of the milk was 10.37% dry matter, 2.7% fat, 3.22% protein, and pH value 6.63. A commercial starter culture containing *Streptococcus thermophilus* and *Lactobacillus delbrueckii* subsp. *bulgaricus* (YO‐MIX 205 LYO, Danisco, France) was used for yoghurt fermentation. The turmeric powder (Knorr, Unilever, Turkey) was purchased from a local market. For lactose hydrolysis, a microbial‐derived β‐galactosidase enzyme (NOLA Fit, Chr. Hansen) was applied.

### 2.2 Preparation of Hawthorn Fruit Puree

Hawthorn fruit puree was prepared according to the method described by Rüzgâr (2022), with minor modifications. Seasonally harvested hawthorn fruits were obtained from the vicinity of Ayaş, Ankara, Turkey (40°13′16″ N, 32°30′25″ E). The fruits were washed, mixed with 35% (w/v) water, and cooked in a domestic pressure cooker for 10 min. Domestic pressure cookers typically operate at 0.7–1.0 bar above atmospheric pressure, corresponding to an internal temperature of approximately 116°C–121°C. After cooking, the fruits were rapidly cooled in an ice bath, passed through a fine sieve to remove skins and seeds, and homogenized into a puree. The puree was portioned and stored at −18°C until use in yoghurt production.

### 2.3 Yoghurt Manufacturing

For yoghurt production, a total of 20 L of raw cow's milk was used, of which 4 L was allocated for the control yoghurt sample (YC, non‐hydrolyzed milk), and 16 L was reserved for the production of lactose‐hydrolyzed samples. The principle of lactose‐hydrolyzed dairy products is based on the enzymatic hydrolysis of lactose into glucose and galactose by β‐galactosidase enzyme (Pereira et al. 2020; Facioni et al. 2020). For this purpose, the hydrolysis procedure in the standardized raw cow's milk was performed using the supplier's calculation software (LactoSense, Chr. Hansen). Based on preliminary trials, lactose hydrolysis was carried out at refrigerator temperature for 12 h using NOLA Fit 5500 lactase enzyme (Chr. Hansen) at a dosage corresponding to approximately 12,500 BLU/L milk (equivalent to 0.2% v/v), which ensured complete lactose hydrolysis. The degree of lactose hydrolysis was determined using a rapid portable biosensor system (DirectSens, Chr. Hansen), based on an enzymatic amperometric sensor that quantitatively detects residual lactose. The extent of hydrolysis was evaluated according to the manufacturer's instructions to verify complete lactose hydrolysis (detection limit < 0.01%).

**FIGURE 1:**
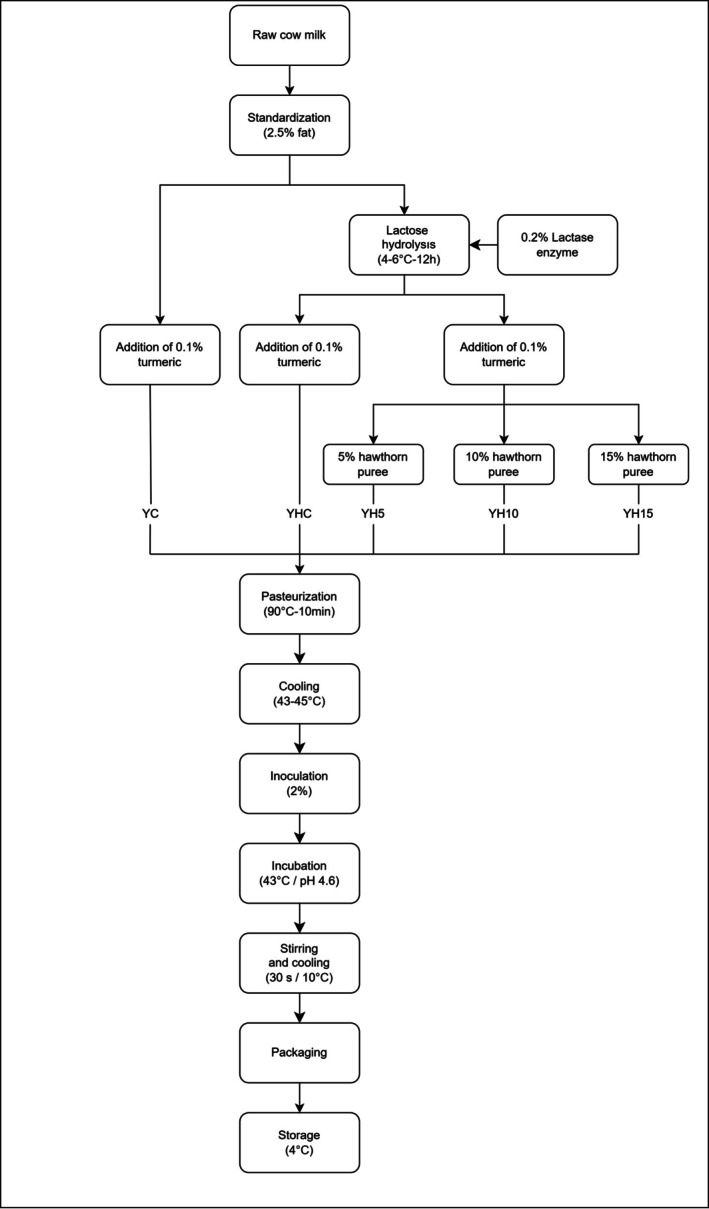
Yoghurt production flow chart.

Following hydrolysis, the milk was divided into four equal proportions, and according to the formulation described in Table 1, hawthorn fruit puree and turmeric powder were incorporated to prepare mixtures for a total of five yoghurt samples, including one control. All mixtures were heat treated at 90°C for 10 min and then cooled to 43°C. Then, 2% (w/v) of the starter culture was inoculated and incubated at 43°C for 4–5 h until the pH reached 4.6. After incubation, the yoghurt samples were homogenized using a laboratory homogenizer (Ultra‐Turrax, model T25 basic; IKA Werke, Staufen, Germany) for 30 s, rapidly cooled to 10°C, and then transferred into 1000 g plastic containers. Samples were stored at +4°C for 21 days (Figure 1). Analyses were performed on days 1, 7, 14, and 21 of storage.

**TABLE 1:** Yoghurt formulations.

Samples	Description
YC	Cow milk +0.1% turmeric powder
YHC	Lactose‐hydrolyzed milk +0.1% turmeric powder
YH5	Lactose‐hydrolyzed milk +5% Hawthorn +0.1% turmeric powder
YH10	Lactose‐hydrolyzed milk +10% Hawthorn +0.1% turmeric powder
YH15	Lactose‐hydrolyzed milk +15% Hawthorn +0.1% turmeric powder

### 2.4 Fermentation Kinetics

Changes in pH were continuously monitored during fermentation using a pH meter equipped with a glass‐electrode (Ohaus, Starter 300, USA). The pH at time zero was measured before the start of fermentation and at 60‐min intervals until pH 4.6. The acidification rate (*V*_max_) was calculated as the time variation of pH (dpH/dt) and expressed as pH units. At the end of the incubation, the following kinetic parameters were calculated: (i) *t*_max_ (h), time at which *V*_max_ was reached; (ii) *t*_5.0_ (h), time at pH 5.0; and (iii) *t*_end_ (h), time to complete the fermentation (Adepoju and Selezneva 2024).

### 2.5 Physicochemical Analyses

In raw milk and yoghurt samples, fat content was determined by the Gerber method, titratable acidity was measured according to the method described by (Hooi et al. 2004), dry matter content was determined gravimetrically, and total protein content was measured using the Kjeldahl method (Hayaloğlu and Özer 2011). The pH value was measured with a glass‐electrode digital pH meter (Ohaus, Starter 300, USA). The water‐holding capacity (WHC%) of yoghurt samples was determined by weighing 20 g of sample, centrifuging at 3000 *g* for 25 min at +4°C, and calculating according to the following equation (Equation 1) (Sodini et al. 2005).

(1) $$\mathrm{WHC}\;\left(\%\right)=\left(W1/W2\;\right)\times 100$$

where W1, Pellet; W2, Sample.

The color properties of the samples were measured using a Konica Minolta CR‐410 colorimeter (Sensing Inc., Osaka, Japan), and each measurement was performed in triplicate. During measurement, the device was set to the International Commission on Illumination (CIE) Lab* color system, recording lightness [L*, ranging from 0 (black) to 100 (white)], redness [a*, red (+*a*) to green (−*a*)], and yellowness [b*, yellow (+*b*) to blue (−*b*)] values. Using the L*, a*, and b* values, chroma [(*a* ^2^ + *b* ^2^)^0^·^5^] was calculated to determine color saturation, and hue angle [tan^−1^(b*/a*)] was calculated to determine color tone (Sanli et al. 2025).

### 2.6 Determination of Total Phenolic Content and Antioxidant Activity

Extracts used for the determination of total phenolic content and antioxidant activity were obtained following the method described by (Hayaloğlu and Özer 2011). For this purpose, 10 g of sample was weighed and centrifuged (Hettich 320 R, Tuttlingen, Germany) at 12,000 *g* for 20 min at +4°C. The resulting filtrate was passed through Whatman No. 42 filter paper and stored at −18°C until analysis.

The total phenolic content (TPC) was determined using the Folin–Ciocalteu method as described by Bengi et al. (2023). Briefly, 300 μL of extract was mixed with 1.5 mL of Folin–Ciocalteu reagent (diluted 1:10 with distilled water) and kept in the dark for 5 min. Then, 1.2 mL of 20% sodium carbonate solution (7.5 g/mL) was added. The mixture was vortexed for 1 min at room temperature, incubated in the dark for 2 h, and the absorbance was measured at 760 nm using a UV–spectrophotometer (Lambda 25 UV/Vis, PerkinElmer, Singapore). The total phenolic content was calculated using a standard curve prepared with gallic acid at different concentrations, and the results were expressed as gallic acid equivalents (mg GAE/g sample).

The antioxidant activity of the samples was determined using two different methods: DPPH (2,2‐diphenyl‐1‐picrylhydrazyl) and ABTS (2,2′‐azino‐bis(3‐ethylbenzothiazoline‐6‐sulfonic acid)). For the determination of antioxidant activity by the DPPH method, 250 μL of sample extract was mixed with 3 mL of 60 μM DPPH solution prepared in ethanol. The mixture was kept in the dark for 30 min, after which the absorbance was measured at 517 nm using a spectrophotometer (Lambda 25 UV/Vis, PerkinElmer, Singapore). The percentage of DPPH inhibition was calculated using the following equation (Equation 2) (Brand‐Williams et al. 1995).

(2) $$\text{DPPH}\%=\frac{A_{\text{blank}}-A_{\text{sample}}}{A_{\text{blank}}}*100$$

For the ABTS assay, a solution containing 7.0 mM ABTS and 2.45 mM potassium persulfate was prepared and kept in the dark at room temperature for 12–16 h to generate the ABTS^·+^ radical. The ABTS^·+^ solution was then diluted with 80% ethanol to obtain an absorbance of 0.700 ± 0.02 at 734 nm. After that, 100 μL of sample extract was added to 2.9 mL of the ABTS solution, vortexed, and incubated in the dark for 30 min. The absorbance was measured at 734 nm, and the antioxidant capacity was calculated based on a standard curve prepared with Trolox at different concentrations. Results were expressed as mg Trolox equivalents per g of sample (mg TE/g) (Kose and Ocak 2020).

### 2.7 Determination of Reological Properties

The rheological properties of the yoghurt samples were analyzed using a Malvern Kinexus Pro+ rheometer (Worcestershire, UK) equipped with a cone probe at a 4° angle (CP4/40SR 239,355). The gap between the probe and the plate was set at 1 mm, and measurements were carried out at +4°C within a shear rate range of 0.1–300 s^−1^. The suitability of the samples to non‐Newtonian flow behavior was determined using the Power Law model, with correlation coefficients (*R* ^2^ > 0.99). All rheological measurements were performed in triplicate.

### 2.8 Determination of Textural Properties

The texture properties of the yoghurt samples were determined using a Texture Analyzer TA‐XT Plus (Stable Micro Systems, UK) equipped with a 5 kg load cell by means of the back‐extrusion test (Herrera et al. 2023). Approximately 100 g of sample contained in plastic cups (50 mm diameter, 50 mm height) was subjected to the test using a 35 mm disc (A/BE‐d35, Stable Micro Systems, UK). During the analysis, the penetration distance, penetration speed, and return speed were set at 30 mm, 1 mm/s, and 10 mm/s, respectively. All measurements were performed in triplicate. The firmness and consistency parameters were recorded using Exponent software (Version 6.1.16.0, Stable Micro Systems).

### 2.9 Determination of Sensorial Properties

The sensory properties of the yoghurt samples were evaluated by a semi‐trained panel of 20 members (12 female, 8 male, aged 19–48 years), consisting of academic staff and students from the Department of Dairy Technology, Faculty of Agriculture, Ankara University. The yoghurt samples were assessed for appearance, flavor, texture, odor, and overall acceptability using a 9‐point hedonic scale ranging from 1 (“dislike extremely”) to 9 (“like extremely”) (Clark and Costello 2016).

The sensory evaluation was conducted with adult volunteers on a voluntary basis and involved a non‐invasive tasting of yoghurt samples prepared from standard food ingredients, posing no known health risks. All participants were informed about the nature of the study and provided verbal informed consent prior to participation.

### 2.10 Statistical Analysis

Statistical analysis was conducted using SPSS 14.01 (License No: 9869264). The yoghurt production process and all experimental analyses were performed in duplicate (*n* = 2). Results are expressed as mean ± standard deviation.

One‐way analysis of variance (ANOVA) was used to evaluate differences among yoghurt samples for gross composition parameters. For storage‐related parameters, two‐way ANOVA was performed to determine the effects of sample type and storage time. When significant differences were detected, means were separated using Tukey's multiple comparison test at a significance level of *p* < 0.05.

## 3 Results and Discussion

### 3.1 Gross Composition

The dry matter, total protein, and fat values of the yoghurt samples are presented in Table 2. The contents of dry matter, protein, and fat ranged between 10.37%–11.19%, 2.82%–3.32%, and 2.2%–2.7%, respectively. As expected, dry matter content increased proportionally with the amount of hawthorn puree added (*p* < 0.05), reflecting the additional solids introduced by the fruit matrix.

**TABLE 2:** Gross chemical composition of yoghurt samples.

Characteristic (g/100 g)	Yoghurt samples				
	YC	YHC	YH5	YH10	YH15
Dry matter	10.37 ± 04^b^	10.94 ± 0.35^ab^	10.63 ± 0.03^ab^	11.06 ± 0.27^a^	11.19 ± 0.28^a^
Total protein	3.32 ± 0.31^a^	3.25 ± 0.06^a^	3.24 ± 0.22^a^	2.89 ± 0.04^b^	2.82 ± 0.005^b^
Fat	2.7 ± 0.06^a^	2.5 ± 0.1^b^	2.3 ± 0.06^c^	2.3 ± 0.05^c^	2.2 ± 0.03^c^

*Note:* Results are expressed as mean ± standard deviation (*n* = 2). Means sharing the same letter within a row are not significantly different (*p* < 0.05).

Abbreviations: YC, cow milk +0.1% turmeric powder; YH5, lactose‐hydrolyzed milk +5% hawthorn puree +0.1% turmeric powder; YH10, lactose‐hydrolyzed milk +10% hawthorn puree +0.1% turmeric powder; YH15, lactose‐hydrolyzed milk +15% hawthorn puree +0.1% turmeric powder; YHC, lactose‐hydrolyzed milk +0.1% turmeric powder.

In contrast, the addition of hawthorn puree led to a reduction in both protein and fat contents compared to the control samples. However, the effect differed between these two components. While even 5% pure incorporation resulted in a significant decrease in fat content (*p* < 0.05), protein content remained statistically unchanged at this level and decreased significantly only at higher incorporation levels (10% and 15%). This differential behavior can be attributed to a dilution effect combined with the compositional characteristics of hawthorn puree. Since the puree contains negligible fat and relatively low protein compared to milk solids, the lower baseline concentration and higher sensitivity of the fat fraction may have led to a more pronounced reduction at lower addition levels. In contrast, the relatively higher initial protein concentration of yoghurt may have buffered minor compositional changes at 5% incorporation. Similar compositional shifts in fruit‐enriched yoghurts have been reported in previous studies (Garofalo et al. 2024). Specifically, the fundamental parameters such as dry matter, protein, and fat contents of the control yoghurt samples (YC and YHC) closely aligned with the typical ranges reported by Ścibisz et al. (2019) for standard cow's milk yoghurt. This consistency in the baseline chemical composition confirms that the initial milk standardization and fermentation processes were successfully executed, thereby providing a reliable reference point for accurately evaluating the specific effects of hawthorn puree and turmeric addition.

### 3.2 Fermentation Kinetics and Acidity

In the study, the *V*_max_ value determined in the YC control sample was 14.83 × 10^−3^ pH/min, whereas in the YHC control sample produced from lactose‐hydrolyzed milk, this value decreased to 12.33 × 10^−3^ pH/min. The yoghurt samples enriched with hawthorn puree (YH5, YH10, and YH15) exhibited even lower *V*_max_ values, which further decreased with increasing puree content, reaching 5.58, 5.17, and 4.83 × 10^−3^ pH/min, respectively. The *t*_max_ values, determined as 3.15–3.00 h in the YC and YHC control samples, were extended in the YH5, YH10, and YH15 samples to 3.45–4.00 h. Similarly, the *t*_5.0_ value, measured as 3 h in the YC control sample, was approximately 4 h in the other samples. The fastest fermentation occurred in the YC sample, which had the highest *V*_max_, with a *t*_end_ value of 4 h. In contrast, the *t*_end_ value increased to 4.55 h in the YHC control produced from lactose‐hydrolyzed milk, and further extended to an average of 5 h in the samples containing hawthorn puree (File S1). Our findings did not agree with those of (Ortiz et al. 2024), who reported that the addition of hawthorn composite (consisting of fruit peel and seeds) reduced the fermentation time of yoghurt by 27%. However, our results were consistent with Wang et al. (2025), who observed a slowing of acidification in yoghurt with increasing levels of hawthorn powder. This deceleration in acid production can be attributed to the specific bioactive and structural components of the fruit. Specifically, phenolic compounds present in hawthorn possess mild antimicrobial properties that can interact with bacterial cell membranes and temporarily stress the starter cultures, thereby extending the fermentation time. Furthermore, the high concentration of complex polysaccharides and pectin in hawthorn significantly increases the viscosity of the milk matrix. This structural change creates a mass transfer limitation, restricting the mobility and diffusion of essential nutrients to the lactic acid bacteria, which ultimately slows down the maximum acidification rate (*V*_max_).

Acidity is a key parameter in determining the quality of fermented dairy products (Güldane 2025). Expressed through pH and titratable acidity, this parameter not only reflects the fermentation activity of the starter culture but also influences product characteristics. The activity of starter cultures varies depending on milk composition, fermentation conditions and substrates (Baniasadi et al. 2022). The pH and titratable acidity values of yoghurt samples during the 21‐day storage period are presented in Table 3. The pH values of the samples ranged between 4.19 and 4.46, while titratable acidity values expressed as lactic acid % varied from 0.80% to 0.91%. During storage, the pH changes in samples enriched with hawthorn fruit puree (YH5, YH10, and YH15) followed a decrease–increase–decrease trend, whereas in the control yoghurt samples (YC and YHC), a consistent decrease was observed (*p* < 0.05). Moreover, the pH values of the YHC sample produced from lactose‐hydrolyzed milk were lower than those of the YC control throughout storage, which may be attributed to the easier metabolism of glucose released during lactose hydrolysis by starter culture bacteria as a readily available energy source (Ibrahim 2018). The LA% values increased in all samples throughout the storage period (*p* < 0.05). However, no difference was detected among the yoghurt samples according to the %LA values determined on different storage days (*p* > 0.05).

**TABLE 3:** pH, lactic acid (%), total antioxidant capacity (DPPH, ABTS), and total phenolic content (TPC) of yoghurt samples.

	Storage (d)	YC	YHC	YH5	YH10	YH15
pH	1	4.46 ± 0.01^Aa^	4.42 ± 0.01^Ba^	4.38 ± 0.02^Ca^	4.37 ± 0.01^Ca^	4.33 ± 0.02^Da^
	7	4.34 ± 0.02^Ab^	4.29 ± 0.02^Bb^	4.30 ± 0.02^Bb^	4.31 ± 0.04^Bb^	4.28 ± 0.01^Bb^
	14	4.27 ± 0.03^Bc^	4.25 ± 0.02^Bc^	4.36 ± 0.03^Aa^	4.37 ± 0.01^Aa^	4.33 ± 0.06^Aa^
	21	4.22 ± 0.04^ABd^	4.25 ± 0.01^Ac^	4.24 ± 0.02^Ac^	4.19 ± 0.04^Bc^	4.19 ± 0.04^Bc^
LA%	1	0.83 ± 0.01^Ac^	0.82 ± 0.01^ABb^	0.82 ± 0.01^ABc^	0.80 ± 0.02^Bc^	0.80 ± 0.01^Bc^
	7	0.86 ± 0.01^Ab^	0.85 ± 0.01 ^ABab^	0.83 ± 0.01^BCbc^	0.83 ± 0.02^BCb^	0.82 ± 0.01^Cbc^
	14	0.86 ± 0.01^ABb^	0.87 ± 0.01^Aa^	0.85 ± 0.01^BCb^	0.84 ± 0.02^Cb^	0.84 ± 0.02^Cb^
	21	0.91 ± 0.01^Aa^	0.86 ± 0.01^Ca^	0.88 ± 0.01^Ba^	0.86 ± 0.02^Ca^	0.89 ± 0.02^ABa^
ABTS (mg TE/g)	1	3.61 ± 0.39^Cb^	3.94 ± 0.01^BCa^	4.99 ± 0.16^Ac^	3.53 ± 0.29^Cbc^	4.09 ± 0.04^Bb^
	7	3.59 ± 0.26^Cb^	3.92 ± 0.25^BCa^	5.81 ± 0.06^Aa^	3.12 ± 0.19^Dc^	4.19 ± 0.33^Bb^
	14	4.04 ± 0.23^Bab^	3.58 ± 0.52^CDa^	5.27 ± 0.07^Ab^	3.73 ± 0.07^Cb^	4.15 ± 0.13^Bb^
	21	4.36 ± 0.23^BCa^	3.71 ± 0.51^CDa^	5.89 ± 0.37^Aa^	4.08 ± 0.03^CDa^	5.03 ± 0.21^Ba^
DPPH%	1	78.5 ± 5.6^Aa^	64.7 ± 0.20^Bb^	79.8 ± 4.60^Aa^	73.9 ± 5.70^Aab^	80.6 ± 1.50^Aa^
	7	83.1 ± 1.8^Aa^	77.6 ± 2.10^Ba^	83.9 ± 1.30^Aa^	58.9 ± 1.40^Dd^	73.1 ± 1.10^Cc^
	14	81.5 ± 0.2^Aa^	77.7 ± 3.80^Ba^	77.9 ± 0.70^Bab^	78.0 ± 2.10^Ba^	78.4 ± 0.90^Bb^
	21	63.4 ± 2.3^Cb^	64.1 ± 2.20^Cb^	75.9 ± 1.40^Ab^	66.3 ± 0.30^Cbc^	72.9 ± 0.00^Bc^
TPC (mg GAE/g)	1	3.58 ± 0.06^Bb^	4.12 ± 0.33^Aa^	3.95 ± 0.01^Ab^	3.10 ± 0.05^Cc^	3.56 ± 0.14^Bb^
	7	3.53 ± 0.03^Bb^	3.87 ± 0.38^Ba^	3.86 ± 0.05^Bb^	3.74 ± 0.17^Bb^	4.43 ± 0.28^Aa^
	14	3.28 ± 0.13^Cc^	3.93 ± 0.22^ABa^	3.38 ± 0.29^Cc^	3.79 ± 0.05^Bb^	4.05 ± 0.24^Aa^
	21	4.10 ± 0.17^Aa^	4.31 ± 0.30^Aa^	4.43 ± 0.09^Aa^	4.42 ± 0.02^Aa^	4.28 ± 0.48^Aa^

*Note:* Results are expressed as mean ± standard deviation (*n* = 2). Different uppercase letters within the same storage day indicate significant differences among yoghurt samples (*p* < 0.05). Different lowercase letters within the same column indicate significant differences during storage (*p* < 0.05).

Abbreviations: ABTS, 2,2′‐azino‐bis (3‐ethylbenzothiazoline‐6‐sulfonic acid) radical scavenging activity; DPPH, 2,2‐diphenyl‐1‐picrylhydrazyl radical scavenging activity; LA, Lactic acid; TPC, Total phenolic content; YC, cow milk +0.1% turmeric powder; YH5, lactose‐hydrolyzed milk +5% hawthorn puree +0.1% turmeric powder; YH10, lactose‐hydrolyzed milk +10% hawthorn puree +0.1% turmeric powder; YH15, lactose‐hydrolyzed milk +15% hawthorn puree +0.1% turmeric powder; YHC, lactose‐hydrolyzed milk +0.1% turmeric powder.

It has been reported that in fruit‐enriched yoghurt samples, starter culture bacteria can metabolize fibers and other components derived from the fruit, thereby enhancing their activity and leading to increased acidity (Pereira et al. 2024). However, there are also studies indicating that trends in acidity may increase and decrease depending on the natural acidity of the fruit, its sugar composition, and the interaction of starter cultures. In agreement with our findings, (Carmona et al. 2021) observed fluctuations in pH values during storage in yoghurts containing prickly pear. (Buchilina and Aryana 2021) reported that the addition of monk fruit to camel milk yoghurt led to a decrease in pH during storage, while no changes were observed in titratable acidity. Similarly, (Fitratullah et al. 2019) found that the incorporation of red dragon fruit caused a decrease in pH values proportional to the fruit concentration, whereas (Hertanto and Pramono 2019) determined that avocado pulp addition had no effect on acidity.

### 3.3 Antioxidant Capacity and Phenolic Compounds

The choice of analytical method and the type of radical or oxidant species used to determine antioxidant capacity can markedly influence the results obtained. Accordingly, different assays may produce distinct activity rankings for the same compounds (Starowicz and Zieliński 2019; Herrera et al. 2023). Therefore, in the present study, two widely applied methods in dairy research, namely ABTS and DPPH assays, were employed to provide a broader evaluation of antioxidant capacity.

The antioxidant activity values (ABTS and DPPH) and total phenolic content (TPC) of the yoghurt samples during storage are presented in Table 3. In the hawthorn puree–enriched samples (YH5, YH10, and YH15), ABTS values ranged from 3.12 to 5.89 mg TE/g, whereas the control samples (YC and YHC) ranged between 3.58 and 4.36 mg TE/g. Regarding the control samples, different behaviors were observed during storage. While ABTS values of the lactose‐hydrolyzed sample (YHC) remained statistically stable throughout storage (*p* > 0.05), the non‐hydrolyzed control (YC) exhibited a significant increase by day 21 compared to the initial storage days (*p* < 0.05).

In contrast, all hawthorn puree–enriched samples showed significant variations during storage (*p* < 0.05). These changes were characterized by initial fluctuations followed by higher ABTS values toward the end of the storage period. Numerically, the highest ABTS value (5.89 mg TE/g) was recorded in YH5 on day 21. The increase observed during storage may be associated with progressive release of bound phenolic compounds or structural modifications within the yoghurt matrix that enhance radical scavenging capacity over time.

Similarly, DPPH values of the hawthorn‐enriched yoghurts ranged from 58.9% to 83.9%, while the control samples varied between 63.4% and 83.1%. Although enrichment with hawthorn contributed to antioxidant activity, the control sample YC also exhibited relatively high DPPH values throughout storage, in some cases comparable to those of enriched samples. This finding suggests that turmeric powder, incorporated into all formulations, substantially contributed to the overall antioxidant potential (Buniowska‐Olejnik et al. 2025).

The higher antioxidant activity observed in fruit‐containing yoghurts is generally attributed to polyphenolic compounds (Priyashantha et al. 2025). The antioxidant properties of plant materials are strongly associated with phenolic hydroxyl groups. During storage, structural modifications of polyphenols and possible release of bound phenolics may enhance measurable antioxidant capacity in dairy matrices (Tami et al. 2022; Okur 2023; Pereira et al. 2024). In the present study, TPC values showed fluctuations during storage; however, numerically higher values were observed on day 21 across all sample groups.

Overall, ABTS, DPPH, and TPC values exhibited non‐linear changes throughout storage, reflecting dynamic interactions between milk proteins and polyphenols introduced via turmeric and hawthorn puree (Oliveira et al. 2015; Garofalo et al. 2024). Notably, increasing the proportion of hawthorn puree did not proportionally increase antioxidant activity. Instead, the YH5 sample (5% hawthorn) frequently demonstrated antioxidant levels comparable to or higher than those of YH10 and YH15, suggesting possible matrix saturation effects or polyphenol–protein interactions limiting extractability at higher enrichment levels.

Regarding lactose hydrolysis, although YC showed a significant increase in ABTS values by the end of storage, overall differences between YC and YHC were limited. However, differences in DPPH and TPC values between these samples on certain storage days may be linked to lactose hydrolysis. Hydrolysis of lactose produces glucose and galactose, which exhibit greater reducing capacity than lactose. Because the Folin–Ciocalteu assay is not entirely specific to phenolic compounds and can also respond to reducing substances, the higher TPC values detected in YHC may partially reflect increased reducing sugar content rather than a genuine increase in phenolic concentration.

Collectively, the findings confirm that turmeric powder plays a major role in enhancing antioxidant capacity (Buniowska‐Olejnik et al. 2025), while hawthorn enrichment contributes variably depending on storage time and matrix interactions.

### 3.4 Rheological Properties

The flow behavior properties of the yoghurt samples during storage are presented in Table 4. The power Law model successfully described the flow behavior of all yoghurt samples. The flow behavior index (*n*), used to evaluate the similarity of flow to Newtonian behavior, is considered pseudoplastic when *n* < 1 in non‐Newtonian systems (Zhang et al. 2025). In the yoghurt samples (YC, YHC, YH10, and YH15), the flow behavior index (n) values were determined to range between 0.13 and 0.16 at the beginning of storage, decreasing to between 0.10 and 0.11 by the end of storage, indicating pseudoplastic flow behavior. However, the n values of the YH5 sample remained constant throughout storage. Low n values reflect greater deviation from Newtonian flow behavior, indicating a higher degree of pseudoplasticity (Priyashantha et al. 2025). The reduction in this rheological parameter may be attributed to the rearrangement of gel particles (Şanlı et al. 2026). Changes observed in flow parameters are associated with chemical interactions resulting from proportional differences between biopolymer types and the carbohydrate and protein components present in the samples (Priyashantha et al. 2025). While significant differences were observed among the n values of the samples on Day 1 (*p* < 0.05), these differences were not significant on the subsequent storage days (*p* > 0.05).

**TABLE 4:** Firmness, consistency, shear viscosity, flow behavior index (*n*), consistency index (*K*), and WHC% of yoghurt samples.

	Storage (d)	YC	YHC	YH5	YH10	YH15
Firmness (g)	1	20.54 ± 0.52^Aab^	18.98 ± 0.00^Ba^	16.15 ± 0.03^Da^	17.19 ± 1,45^Ca^	15.78 ± 0.13^Eb^
	7	20.93 ± 1.39^Aab^	18.86 ± 1.54^ABa^	16.40 ± 1.41^Ba^	19.10 ± 3.60^Aa^	15.77 ± 1.23^Bb^
	14	19.85 ± 0.12^Ab^	17.68 ± 0.63^Ba^	15.35 ± 0.65^Ca^	16.13 ± 1.79^BCa^	13.88 ± 0.28^Dc^
	21	21.61 ± 0.33^Aa^	18.61 ± 0.70^Ba^	16.10 ± 0.74^Ca^	16.81 ± 1.79^BCa^	16.36 ± 0.48^Ca^
Consistency (g.s)	1	546.09 ± 24^Aa^	495.96 ± 40^Aa^	415.55 ± 20^Ba^	433.13 ± 39^ABa^	404.43 ± 30^Ba^
	7	560.98 ± 46^Aa^	503.01 ± 48^ABa^	435.94 ± 42^BCa^	511.93 ± 102^ABa^	416.93 ± 37^Ca^
	14	511.91 ± 40^Aa^	457.33 ± 50^Aa^	397.06 ± 17^Ba^	418.57 ± 47^ABa^	380.37 ± 10^Ba^
	21	555.89 ± 70^Aa^	480.33 ± 17^ABa^	416.18 ± 18^Ca^	433.41 ± 51^BCa^	419.14 ± 13^Ca^
Sheer Viscosity (Pa.s)	1	0.45 ± 0.04^Cc^	0.62 ± 0.09^Ba^	0.79 ± 0.03^Aa^	0.72 ± 0.06^Ac^	0.77 ± 0.33^Ab^
	7	0.53 ± 0.07^Db^	0.62 ± 0.00^Ca^	0.69 ± 0.15^Cb^	1.65 ± 0.12^Aa^	1.35 ± 0.13^Ba^
	14	0.47 ± 0.04^Cc^	0.34 ± 0.02^Dc^	0.32 ± 0.00^Dd^	0.86 ± 0.01^Ab^	0.79 ± 0.13^Bb^
	21	0.68 ± 0.06^BCa^	0.53 ± 0.06^Cb^	0.55 ± 0.12^Cc^	0.77 ± 0.22^Bbc^	1.39 ± 0.47^Aa^
*n*	1	0.16 ± 0.01^Aa^	0.13 ± 0.01^Ba^	0.12 ± 0.01^Ba^	0.13 ± 0.01^Ba^	0.13 ± 0.01^Ba^
	7	0.12 ± 0.01^Ab^	0.13 ± 0.00^Aa^	0.12 ± 0.01^Aa^	0.12 ± 0.01^Aab^	0.11 ± 0.01^Ab^
	14	0.11 ± 0.01^Ab^	0.11 ± 0.01^Ab^	0.12 ± 0.01^Aa^	0.11 ± 0.00^Ab^	0.13 ± 0.01^Aa^
	21	0.11 ± 0.01^Ab^	0.11 ± 0.01^Ab^	0.12 ± 0.00^Aa^	0.10 ± 0.01^Ab^	0.11 ± 0.00^Ab^
K (Pa.s^*n*^)	1	27.6 ± 1.51^Ba^	24.5 ± 1.68^Bb^	21.6 ± 0.07^Cc^	23.6 ± 0.55^Bc^	40.3 ± 7.95^Aa^
	7	26.2 ± 2.03^Ba^	22.7 ± 0.00^Cc^	26.3 ± 2.11^Bb^	42.2 ± 6.75^Aa^	29.9 ± 3.15^Bb^
	14	27.9 ± 1.79^Ca^	36.6 ± 1.49^Ba^	38.9 ± 0.37^Aa^	21.6 ± 0.03^Ed^	22.5 ± 0.49^Dc^
	21	22.6 ± 0.63^Cb^	26.2 ± 1.98^Bb^	28.3 ± 3.79^Bb^	28.3 ± 1.99^Bb^	44.1 ± 11.80^Aa^
WHC%	1	34.80 ± 0.55^Cb^	35.40 ± 0.26^Ca^	35.20 ± 0.58^Cb^	37.90 ± 1.99^ABa^	39.80 ± 0.82^Aa^
	7	35.20 ± 0.77^CDb^	34.40 ± 0.44^Db^	35.70 ± 0.38^Cab^	37.60 ± 0.63^Ba^	39.40 ± 0.60^Aa^
	14	36.30 ± 0.05^Ba^	34.30 ± 0.40^Db^	35.20 ± 0.31^Cb^	37.20 ± 0.96^Aa^	38.20 ± 0.09^Ab^
	21	36.40 ± 0.08^Ba^	34.70 ± 0.63^Cab^	36.10 ± 0.30^Ba^	38.80 ± 0.07^Aa^	39.60 ± 0.73^Aa^

*Note:* Results are expressed as mean ± standard deviation (*n* = 2). Different uppercase letters within the same storage day indicate significant differences among yoghurt samples (*p* < 0.05). Different lowercase letters within the same column indicate significant differences during storage (*p* < 0.05).

Abbreviations: YC, cow milk +0.1% turmeric powder; YH5, lactose‐hydrolyzed milk +5% hawthorn puree +0.1% turmeric powder; YH10, lactose‐hydrolyzed milk +10% hawthorn puree +0.1% turmeric powder; YH15, lactose‐hydrolyzed milk +15% hawthorn puree +0.1% turmeric powder; YHC, lactose‐hydrolyzed milk +0.1% turmeric powder.

The consistency index (*K*) values of the yoghurt samples during storage ranged from 21.6 to 44.1 Pa·s^*n*^, showing both increasing and decreasing trends over time. Among the samples, only the yoghurt containing 15% hawthorn puree exhibited a decrease in K values on Days 7 and 14, which subsequently increased by the end of storage, reaching the highest value of 44.1 Pa·s^*n*^ (*p* < 0.05). The shear viscosity values, initially measured at 0.45–0.79 Pa·s at the beginning of storage, increased to 0.53–1.39 Pa·s by the end of the storage period. These values also showed fluctuating patterns throughout storage (*p* < 0.05).

The shear viscosity values of hawthorn puree–enriched samples (YH5, YH10, and YH15) were significantly higher than those of the control samples (YC and YHC) (*p* < 0.05). While the shear viscosity values of yoghurts containing different concentrations of hawthorn puree were initially similar (0.72–0.79 Pa·s) on the first day of storage, the YH15 sample exhibited notable dynamic changes over time. Specifically, its shear viscosity increased significantly by Day 7, decreased on Day 14, and increased again toward the end of storage, ultimately reaching the highest overall value (1.39 Pa·s) on Day 21.

These fluctuations can be attributed to ongoing structural rearrangements within the yoghurt gel during storage. The overall enhanced viscosity can be primarily attributed to the gelling property of pectin present in hawthorn. As Wang et al. (2025) noted, hawthorn pectin can fill the pores by forming a colloidal network through interactions with milk proteins, thereby enhancing the elasticity, consistency, and stability of the gel matrix. Within this context, the initial viscosity increase up to day 7 reflects the progressive strengthening of protein–pectin interactions under acidic conditions. The subsequent decrease on Day 14 may be related to post‐acidification, localized weakening of the protein–polysaccharide network, and possible serum redistribution within the matrix. Finally, the increase observed on Day 21 is likely associated with further stabilization and progressive reorganization of the gel structure, resulting in enhanced water immobilization within the network.

### 3.5 Textural Properties

The firmness and consistency values of the yoghurts determined during storage are presented in Table 4. Firmness, an important texture parameter in yoghurt, corresponds to the force required for product deformation (Dimitrellou et al. 2025). This parameter reflects the structural properties of the yoghurt gel, where higher values indicate greater gel strength and stability (Feng et al. 2019). In this study, the YC control yoghurt sample (produced from non‐hydrolyzed milk) exhibited the highest firmness values (20.54–21.61 g). In contrast, the YHC control sample (produced from lactose‐hydrolyzed milk without hawthorn puree) showed lower firmness values (17.68–18.98 g). This reduction in firmness can be attributed to the structural modifications induced by lactose hydrolysis. The enzymatic breakdown of lactose into glucose and galactose alters the carbohydrate profile of milk, which may influence fermentation dynamics and modify protein network formation during gelation.

Specifically, increased levels of monosaccharides may promote the growth of *Lactobacillus delbrueckii* subsp. bulgaricus, potentially leading to higher production of extracellular polysaccharides (EPS). These EPS can modify protein–protein interactions and water distribution within the yoghurt matrix, contributing to a softer gel structure and reduced curd firmness (Ichimura and Ichiba 2024). Consistently, Ichimura and Ichiba (2024) reported that yoghurt samples with ≥ 50% lactose hydrolysis exhibited significantly reduced gel firmness. Similarly, Biçer et al. (2025) observed that yoghurts produced from lactose‐hydrolyzed cow's milk showed lower consistency values.

Furthermore, enrichment with hawthorn puree led to a significant decrease in both firmness and consistency values of the yoghurt samples (*p* < 0.05). However, with the exception of the YH15 sample, storage time did not have a significant effect on firmness (*p* > 0.05).

This reduction may be attributed to structural and compositional modifications induced by fruit incorporation. The addition of hawthorn puree may reduce the effective protein concentration in the continuous phase and alter total solid composition, thereby weakening the three‐dimensional casein gel network. In addition, fruit‐derived organic acids may influence gel microstructure and modify protein aggregation behavior during fermentation.

Moreover, hawthorn is rich in phenolic compounds, which are known to interact with milk proteins through hydrogen bonding and hydrophobic interactions (Wang et al. 2025). These interactions may interfere with protein–protein associations within the gel matrix and alter water distribution, resulting in a less compact structure and reduced mechanical strength. Similar reductions in firmness following fruit pulp addition have been reported by Feng et al. (2019). Likewise, Dimitrellou et al. (2025) reported that the addition of apple pulp did not significantly affect yoghurt hardness (firmness), and only a slight, non‐significant decrease was observed during storage.

In contrast, studies using fruit ingredients in powder or extract form, such as pomegranate peel extract (Jany et al. 2024) and hawthorn powder (Wang et al. 2025), reported increased firmness, which was attributed to higher dry matter content and reinforcement of the gel structure. These differences suggest that the physical form and compositional characteristics of the fruit ingredient play a crucial role in determining yoghurt texture.

As shown in Table 4, although numerical fluctuations were observed, no statistically significant differences were detected in the consistency values of each formulation during storage (*p* > 0.05), as indicated by identical lowercase letters within columns. The highest consistency value (560.98 g·s) was recorded in the YC control sample, while the lowest value (380.37 g·s) was observed in the YH15 sample. Overall, lactose hydrolysis and hawthorn puree addition resulted in lower consistency values compared to the YC control.

Among the hawthorn‐enriched yoghurts, the YH10 sample exhibited relatively higher firmness and consistency values compared to YH5 and YH15. It is well established that excessive levels of fiber and polyphenols in fruit yoghurts can disrupt the three‐dimensional structure of the protein gel (Feng et al. 2019; Wang et al. 2025). This interpretation is supported by the observation that the YH15 sample, containing the highest proportion of hawthorn puree (15%), showed the lowest firmness and consistency values.

Additionally, the samples (YH5, YH10, and YH15) that exhibited lower firmness and consistency than the YC control also had comparatively lower protein contents and pH values, which may have negatively influenced their textural properties. These findings are consistent with previous reports indicating that protein content in yoghurt and other fermented dairy products is closely associated with their physicochemical characteristics (Feng et al. 2019; Dimitrellou et al. 2025).

### 3.6 Water Holding Capacity

The water‐holding capacity (WHC) values of the yoghurt samples are presented in Table 4. WHC is one of the most important physical properties determining the quality of yoghurt and other fermented dairy products (Buniowska‐Olejnik et al. 2025; Wang et al. 2025). Factors such as milk composition, starter culture activity, added ingredients, and processing conditions can influence the WHC of the product. Consequently, natural components added for yoghurt enrichment may either increase or decrease WHC depending on their water‐binding properties (Hamed et al. 2021; Tami et al. 2022; Buniowska‐Olejnik et al. 2025). In this study, the WHC values of yoghurt samples ranged between 34.43%–39.80%. From day 14 of storage onward, WHC values increased in the control sample (YC) and in the sample containing 5% hawthorn puree (YH5), while a decrease was observed in the control sample produced from lactose‐hydrolyzed milk (YHC) (*p* < 0.05).

A decrease in WHC during storage is a typical phenomenon in yoghurt and has been associated with structural changes in protein complexes and the product's water content. Despite the presence of turmeric powder, which has been reported to have a stabilizing effect on WHC (Buniowska‐Olejnik et al. 2025), the overall WHC of the YHC sample was lower compared to the YC sample. This decline is scientifically attributed to the structural modifications induced by lactose hydrolysis. Specifically, the enzymatic breakdown of lactose into monosaccharides alters the carbohydrate profile, which subsequently modifies protein–protein interactions and fermentation dynamics (Ichimura and Ichiba 2024). As a result, a softer and less cohesive gel network is formed, reducing the matrix's capacity to retain water. Furthermore, an evaluation of the storage period reveals different WHC trends among the samples. While the WHC of the non‐hydrolyzed control (YC) and YH5 samples significantly increased toward the end of storage (Day 21) (*p* < 0.05), the lactose‐hydrolyzed control (YHC) exhibited its highest WHC on the first day, followed by a decrease. Meanwhile, the samples with higher hawthorn puree concentrations (YH10 and YH15) maintained the highest overall WHC values throughout the entire 21‐day period, demonstrating enhanced gel stability.

In the samples containing 10% and 15% hawthorn puree (YH10 and YH15), WHC values were generally higher than those of the other samples. Furthermore, the WHC of the YH10 sample remained completely stable throughout the entire storage period, with no significant changes observed (*p* > 0.05). Similarly, the YH15 sample maintained highly steady WHC levels, exhibiting only a minor fluctuation on Day 14 (*p* < 0.05). The presence of fibers such as pectin, which act as stabilizing and gelling agents in fruits, is known to enhance WHC in fruit yoghurts (Elshynrawy et al. 2024). In this study, a gradual increase in WHC was observed with increasing levels of hawthorn puree addition. Notably, while the YH15 sample exhibited the highest numerical WHC values (38.20%–39.80%) on all storage days, there was no significant difference between the YH10 and YH15 samples on Days 14 and 21 (*p* > 0.05). Our result was found to be consistent with Wang et al. (2025), who determined higher WHC values in yoghurt with increasing hawthorn addition. This effect is primarily attributed to the high pectin content of hawthorn fruit, which binds water molecules within the protein network. In addition, the combined use of turmeric powder and hawthorn puree resulted in a more pronounced improvement in yoghurt WHC compared to their individual addition. This phenomenon can be explained by a synergistic structural interaction; the diverse dietary fibers from turmeric and the pectin from hawthorn collaboratively interact with the casein network. This multi‐component interaction forms a denser, highly cross‐linked colloidal matrix that immobilizes the aqueous phase much more effectively than a single polysaccharide source (Buniowska‐Olejnik et al. 2025; Wang et al. 2025).

### 3.7 Color Properties

Color is an important quality attribute that influences consumer perception and overall acceptability of foods (Ścibisz et al. 2019). The changes in L*, a*, b*, Chroma, and Hue Angle values describing the color characteristics of yoghurt samples during storage are presented in Table 5. In the yoghurt samples enriched with hawthorn puree (YH5, YH10, and YH15), the measured L* values significantly decreased (*p* < 0.05) as the puree concentration increased. This result explains the darker color tones observed in hawthorn‐enriched samples compared to the control samples (YC and YHC). These findings are consistent with those of (Wang et al. 2025), who reported a gradual decrease in L* values with increasing levels of hawthorn powder in yoghurt. In the control samples (YC and YHC), no significant changes in L* values were observed during storage (*p* > 0.05), indicating stable brightness.

**TABLE 5:** L*, a*, b*, chroma, and hue angle values representing the color properties of yoghurt samples.

	Storage (d)	YC	YHC	YH5	YH10	YH15
L*	1	90.14 ± 0.42^Ab^	89.21 ± 0.15^Aa^	87.29 ± 0.32^Ba^	84.95 ± 0.29^Cb^	83.71 ± 0.30^Dab^
	7	89.04 ± 0.69^Ab^	88.45 ± 0.58^Aa^	86.66 ± 0.13^Bb^	84.79 ± 0.14^Cb^	83.09 ± 0.14^Db^
	14	90.29 ± 1.06^Aab^	89.85 ± 0.95^Aa^	86.25 ± 0.87^Bb^	85.85 ± 0.21^Ba^	84.22 ± 0.43^Ca^
	21	92.96 ± 1.06^Aa^	89.82 ± 0.95^Ba^	87.86 ± 0.07^Ca^	85.85 ± 0.35^Da^	83.65 ± 0.12^Eab^
a*	1	−13.11 ± 0.04^Da^	−12.99 ± 0.07^Db^	−9.49 ± 0.03^Ca^	−7.03 ± 0.45^Bab^	−4.91 ± 0.01^Aa^
	7	−13.27 ± 0.10^Ca^	−13.22 ± 0.00^Cb^	−9.63 ± 0.06^Ba^	−6.58 ± 0.49^Aa^	−6.18 ± 0.02^Ac^
	14	−12.73 ± 0.10^Da^	−12.55 ± 0.06^Da^	−9.48 ± 0.04^Ca^	−6.99 ± 0.15^Ba^	−5.41 ± 0.18^Ab^
	21	−13.24 ± 0.10^Da^	−13.22 ± 0.06^Db^	−9.75 ± 0.18^Ca^	−7.56 ± 0.12^Bb^	−5.21 ± 0.01^Ab^
b*	1	41.86 ± 0.21^BCc^	42.92 ± 0.41^Aab^	43.07 ± 0.23^Aa^	43.35 ± 1.22^Aa^	41.99 ± 0.08^Bb^
	7	42.47 ± 0.56^Ab^	42.60 ± 0.78^Aab^	42.22 ± 0.14^Ab^	41.96 ± 0.51^Ab^	40.81 ± 0.21^Bc^
	14	42.84 ± 0.17^Ab^	42.71 ± 0.29^Ab^	41.95 ± 0.10^Bc^	42.31 ± 0.56^Aab^	41.99 ± 0.08^Bb^
	21	44.53 ± 0.17^Aa^	43.23 ± 0.31^Ba^	43.02 ± 0.14^Ba^	43.36 ± 0.88^Ba^	42.31 ± 0.09^Ba^
Chroma	1	44.03 ± 0.31^Bb^	44.87 ± 0.41^Aa^	44.19 ± 0.19^Ba^	44.18 ± 0.94^ABa^	42.50 ± 0.21^Cb^
	7	44.54 ± 0.46^Ab^	44.49 ± 0.80^Aab^	43.13 ± 0,048^Bc^	42.54 ± 0.39^Cb^	41.48 ± 0.21^Dc^
	14	44.59 ± 0.03^Ab^	44.37 ± 0.05^Bb^	43.05 ± 0.061^Cc^	43.08 ± 0.57^Cb^	42.10 ± 0.09^Dc^
	21	46.49 ± 0.03^Aa^	45.24 ± 0.13^Ba^	43.85 ± 0.00^Cb^	43.77 ± 0.87^Cab^	43.04 ± 0.05^Ca^
Hue Angle	1	107.2 ± 0.07^Aa^	106.9 ± 0.03^Aa^	102.5 ± 0.15^Ba^	99.2 ± 0.78^Cb^	96.7 ± 0.12^Db^
	7	107.1 ± 0.27^Aa^	107.1 ± 0.29^Aa^	103.1 ± 0.09^Ba^	101.7 ± 0.69^Ba^	98.5 ± 0.00^Da^
	14	106.7 ± 0.09^Aa^	106.6 ± 0.07^Aa^	103.1 ± 0.07^Ba^	99.5 ± 0.06^Cb^	97.5 ± 0.19^Dab^
	21	106.4 ± 0.13^Aa^	106.9 ± 0.07^Aa^	102.9 ± 0.18^Ba^	99.7 ± 0.32^Cb^	96.9 ± 0.21^Db^

*Note:* Results are expressed as mean ± standard deviation (*n* = 2). Different uppercase letters within the same storage day indicate significant differences among yoghurt samples (*p* < 0.05). Different lowercase letters within the same column indicate significant differences during storage (*p* < 0.05).

Abbreviations: YC, cow milk +0.1% turmeric powder; YH5, lactose‐hydrolyzed milk +5% hawthorn puree +0.1% turmeric powder; YH10, lactose‐hydrolyzed milk +10% hawthorn puree +0.1% turmeric powder; YH15, lactose‐hydrolyzed milk +15% hawthorn puree +0.1% turmeric powder; YHC, lactose‐hydrolyzed milk +0.1% turmeric powder.

However, significant changes in a* values occurred throughout storage (*p* < 0.05). Negative a* values, indicating a tendency toward greenness, were observed in all yoghurt samples. In the control samples (YC and YHC), a* values ranged from −12.55 to −13.27, whereas in the hawthorn‐enriched samples (YH5, YH10, and YH15), a* values shifted from −9.75 to −4.91 as the puree concentration increased, reflecting a gradual trend from green toward red. The negative a* values observed in the control samples are in line with the results reported by (Sıçramaz 2025). While no significant changes in a* values were found in the YC sample during storage (*p* > 0.05), a significant change was detected only on day 21 in the YHC sample (*p* < 0.05). The positive b* values, reflecting a tendency toward yellowness, ranged from 41.86 to 44.53 in yoghurt samples. Depending on the concentration of hawthorn puree, some samples showed no significant change in b* values, while others exhibited only minimal variations. This result suggests that the combined presence of turmeric powder and hawthorn contributed to color stability in yoghurt (Buniowska‐Olejnik et al. 2025). The increase in the positively measured b* parameters of the samples, which was determined to be significant (*p* < 0.05) during storage, emphasized that the yellowish tones originating from turmeric powder gradually increased over time.

Chroma values, representing color intensity, ranged between 41.48 and 46.49. Higher Chroma values were measured in the control samples (44.03–46.49), which were comparable to those of YH5 and YH10 (43.05–44.19). However, the lowest Chroma values (41.48–43.04) were observed in the YH15 sample containing the highest concentration of hawthorn puree, indicating a reduction in color intensity (*p* < 0.05) and a shift toward the yellow spectrum. Hue Angle values significantly decreased with increasing hawthorn puree concentration (*p* < 0.05), although no significant changes were observed during storage. This indicates that the characteristic tones derived from turmeric powder in the control samples were more distinct and stable.

Plants contain a wide range of pigments, including chlorophylls, carotenoids, anthocyanins, and flavonoids, which contribute to their characteristic colors (Taneva and Zlatev 2024). Considering that the natural pigments in turmeric powder are more stable under acidic than alkaline conditions (Sıçramaz 2025), the observed changes in yoghurt color properties during storage can be associated with the increasing concentration of hawthorn puree. The presence of natural pigments such as anthocyanins found in hawthorn also changes the surface color with an increase in the pigment concentration incorporated into the yoghurt matrix (Wang et al. 2025).

### 3.8 Sensory Properties

**FIGURE 2:**
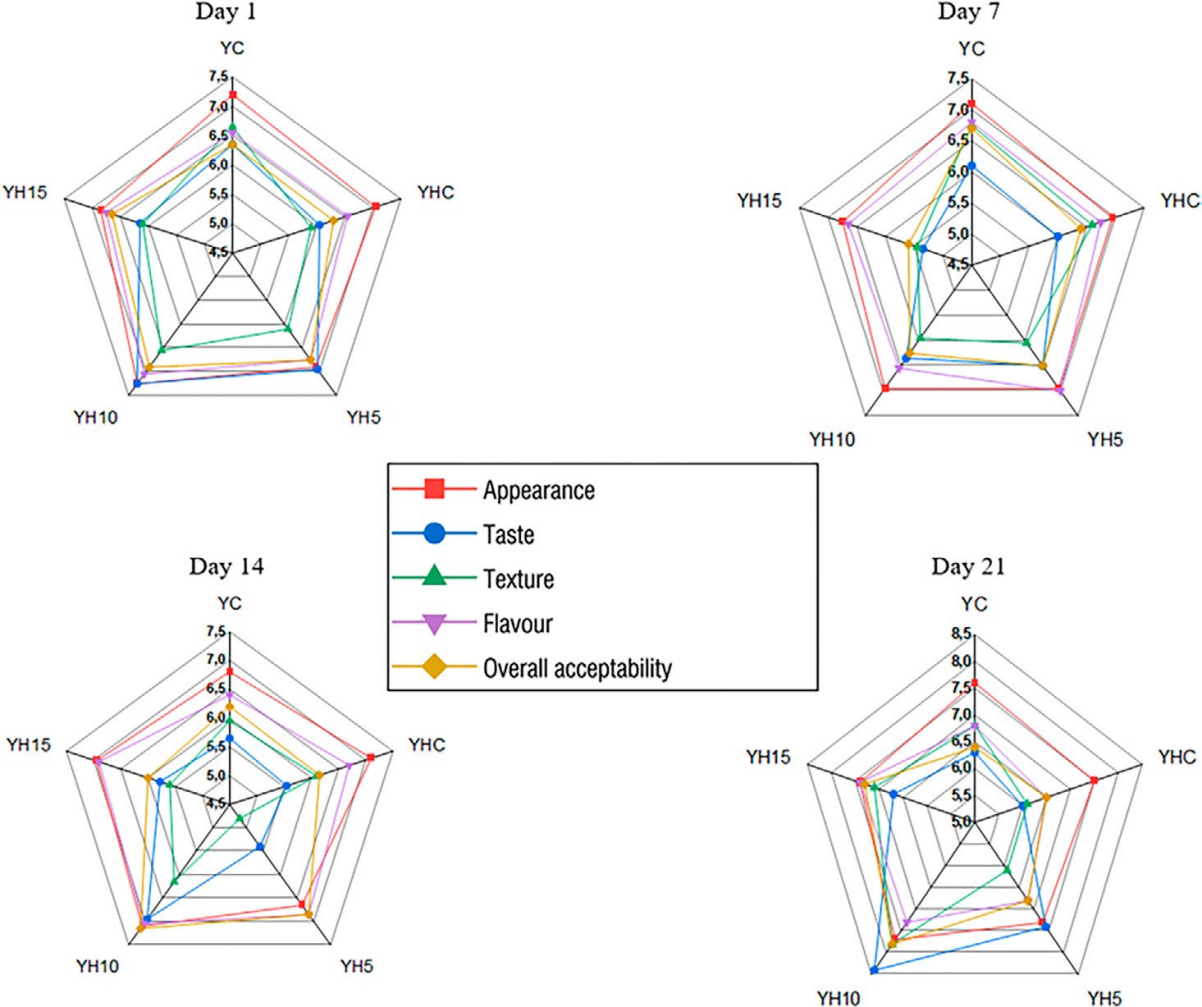
Sensory properties of yoghurt samples.

The study showed that yoghurt samples enriched with hawthorn puree had similar or even more desirable appearance, odor, and taste characteristics than the control samples. In terms of taste, the yoghurt containing 10% hawthorn puree was the most favored by the panelists (Figure 2). These results support the findings of (Utebaeva et al. 2024), who reported that the addition of hawthorn extract enhanced the sensory attributes of a symbiotic fermented dairy product without masking its natural flavor–aroma profile. The researchers also noted that hawthorn extract improved flavor characteristics compared to the control while leaving the consistency unchanged. In contrast, our study revealed that hawthorn puree addition led to a decrease in yoghurt consistency scores. Nevertheless, among all samples, the YH10 yoghurt enriched with 10% hawthorn puree demonstrated superior flavor characteristics on Days 14 and 21 of storage. This improvement in flavor indicates that fruit‐enriched products can enhance sensory attributes and achieve higher consumer acceptability. Consistent with findings from other researchers, it can also be suggested that the appropriate level of turmeric addition contributes positively to the taste and flavor of yoghurt (Liu et al. 2018; Wijesekara et al. 2022; Buniowska‐Olejnik et al. 2025).

### 3.9 Limitations

While the current study provides comprehensive insights into the physicochemical, rheological, and sensory properties of yoghurt enriched with hawthorn puree and turmeric powder, certain limitations should be acknowledged. First, the lack of microbiological analysis restricts our understanding of the specific viability and population dynamics of the starter cultures ( *Lactobacillus delbrueckii* subsp. *bulgaricus* and *Streptococcus thermophilus* ) during the 21‐day storage period. Second, although water‐holding capacity (WHC) was thoroughly evaluated, the absence of direct syneresis (whey expulsion) measurements limits a complete macro‐structural assessment of the gel network's physical stability under mechanical stress. Finally, the study did not include a comprehensive shelf‐life or microbial stability assessment (e.g., detection of yeast, mold, or coliforms), which is crucial for determining the ultimate commercial viability and safety of the product over extended periods. Future studies should address these aspects by incorporating detailed microbiological profiling and extended shelf‐life stability tests to build upon the present findings.

## 4 Conclusion

Enrichment of yoghurt with less commonly known fruits provides synergistic benefits by combining the health‐promoting properties of fruits with the nutritional advantages of fermented dairy products. In the present study, the formulation of a lactose‐hydrolyzed yoghurt with hawthorn puree and turmeric powder demonstrated significant physicochemical and functional improvements. Quantitatively, the addition of hawthorn puree (up to 15%) slowed the maximum acidification rate (*V*_max_) from 14.83 × 10^−3^ pH/min in the control to 4.83 × 10^−3^ pH/min. However, it significantly enhanced the water‐holding capacity (WHC), which reached maximum values of 38.20%–39.80% in the YH15 samples. Furthermore, the enriched yoghurts maintained high antioxidant activity, with ABTS values reaching up to 5.89 mg TE/g. Textural and rheological measurements indicated that enrichment with an optimal level (10%) of hawthorn puree improved the elasticity and consistency of yoghurt gels, thereby achieving the highest sensory acceptability. Furthermore, although fruit‐enriched yoghurts are often expected to have less stable color values, the addition of turmeric powder contributed to greater color stability throughout storage. This finding confirms the role of turmeric powder as a natural colorant with a significant effect on the color parameters of fruit‐based yoghurts. Overall, the findings of this study demonstrate the feasibility of producing an innovative lactose‐hydrolyzed yoghurt, free of added sugars, enriched with hawthorn puree and turmeric powder. Incorporating lesser‐known fruits and spices as functional ingredients may not only enhance the functional properties and consumer satisfaction of yoghurt and related fermented products but also help meet evolving market demands. This offers a highly practical approach for the dairy industry to produce clean‐label, functional products without compromising structural integrity. Nevertheless, certain limitations of this work should be acknowledged, including the absence of specific microbiological viability assays for the starter cultures, the lack of direct syneresis measurements, and the need for comprehensive shelf‐life and microbial stability evaluations. To address these gaps, future research directions should focus on detailed microbiological profiling and extended shelf‐life stability tests. Additionally, future studies integrating diverse probiotic cultures and in vitro digestion models could provide more comprehensive insights into the functional and nutritional impacts of such innovative formulations. Validating the health benefits through in vivo studies and exploring microencapsulation technologies will further support the successful industrial‐scale application of these functional yoghurts.

## Author Contributions

**Muhammed Fidan:** conceptualization, methodology, writing – review and editing, formal analysis. **Tuba Şanlı:** conceptualization, methodology, writing – review and editing, supervision. **Canan Altınay:** conceptualization, methodology, writing – review and editing, formal analysis. **Samet Dönmez:** formal analysis. **Halil İbrahim Öztaş:** formal analysis.

## Funding

This study was supported by the 2209‐A University Student Research Projects Support Program, conducted by the TÜBİTAK Scientist Support Programs Directorate (BİDEB). Project number: 1919B012314100.

## Conflicts of Interest

The authors declare no conflicts of interest.

## Supporting information


**File S1:** Kinetic parameters of lactose‐hydrolyzed yoghurts fortified with hawthorn fruit.

## Data Availability

Even though adequate data has been given in the form of tables and figures, all authors declare that if more data is required then the data will be provided on a request basis.
