# Supplementary material for: Techno‐Functional Properties and Antioxidant Activities of Lactose‐Hydrolyzed Stirred Yoghurts Fortified With Hawthorn Fruit
Source: Food Sci Nutr. 2026 Apr 4;14(4):e71729. doi: 10.1002/fsn3.71729 (PMC13052231; doi:10.1002/fsn3.71729)
Supplement: Supplementary file 1 — File S1: Kinetic parameters of lactose‐hydrolyzed yoghurts fortified with hawthorn fruit. [file FSN3-14-e71729-s001.docx]

**Supplementary File 1** Kinetic parameters of lactose hydrolyzed yoghurts fortified with hawthorn fruit

| **Sample** | **T_end_ (h)** | **V_max_ (10⁻³ pH/min)** | **T_max_ (h)** | **T_5.0_ (h)** |
| --- | --- | --- | --- | --- |
| YC | 4.00 | 14.83 | 3.15 | 3.0 |
| YHC | 4.55 | 12.33 | 3.00 | 4.0 |
| YH5 | 5.20 | 5.58 | 3.45 | 4.2 |
| YH10 | 5.15 | 5.17 | 4.00 | 4.2 |
| YH15 | 5.20 | 4.83 | 4.00 | 4.2 |
